# Disrupted intra- and inter-network connectivity in unilateral acute tinnitus with hearing loss

**DOI:** 10.3389/fnagi.2022.833437

**Published:** 2022-08-01

**Authors:** Gang-Ping Zhou, Wang-Wei Li, Yu-Chen Chen, Heng-Le Wei, Yu-Sheng Yu, Xi Guo, Xindao Yin, Yue-Jin Tao, Hong Zhang

**Affiliations:** ^1^Department of Radiology, The Affiliated Jiangning Hospital of Nanjing Medical University, Nanjing, China; ^2^Department of E.N.T., The Affiliated Jiangning Hospital of Nanjing Medical University, Nanjing, China; ^3^Department of Radiology, Nanjing First Hospital, Nanjing Medical University, Nanjing, China

**Keywords:** functional network connectivity, independent component analysis, resting-state fMRI, acute tinnitus, resting-state network

## Abstract

**Purpose:**

Currently, the underlying neurophysiological mechanism of acute tinnitus is still poorly understood. This study aimed to explore differences in brain functional connectivity (FC) within and between resting-state networks (RSNs) in acute tinnitus patients with hearing loss (ATHL). Furthermore, it also evaluated the correlations between FC alterations and clinical characteristics.

**Methods:**

Two matched groups of 40 patients and 40 healthy controls (HCs) were included. Independent component analysis (ICA) was employed to obtain RSNs and FC differences were calculated within RSNs. In addition, the relationships between networks were conducted using functional network connectivity (FNC) analysis. Finally, an analysis of correlation was used to evaluate the relationship between FNC abnormalities and clinical data.

**Results:**

Results of this study found that seven major RSNs including the auditory network (AN), cerebellum network (CN), default mode network (DMN), executive control network (ECN), sensorimotor network (SMN), ventral attention network (VAN), and visual network (VN) were extracted using the group ICA in both groups. Furthermore, it was noted that the ATHL group showed aberrant FC within the CN, ECN, and VN as compared with HCs. Moreover, different patterns of network interactions were observed between groups, including the SMN-ECN, SMN-CN, ECN-AN, DMN-VAN, and DMN-CN connections. The correlations between functional disconnection and clinical characteristics in ATHL were also found in this study.

**Conclusion:**

In conclusion, this study indicated widespread alterations of intra- and inter-network connectivity in ATHL, suggesting that multiple large-scale network dysfunctions and interactions are involved in the early stage. Furthermore, our findings may provide new perspectives to understand the neuropathophysiological mechanism of acute tinnitus.

## Introduction

Tinnitus is an auditory symptom characterized by the perception of sound without the presence of a corresponding external sound source (Elgoyhen et al., 2015). It has been found that approximately 25% of the adult population experience one or more episodes of acute tinnitus, daily or permanently by 8% (Kandeepan et al., 2019). Although some audiological or psychological interventions such as hearing aids, sound therapy, cognitive behavioral therapy, or counseling and education are helpful for people suffering from tinnitus, a majority of patients with tinnitus are not cured and are seeking a treatment that would provide permanent relief (Langguth et al., 2013). Therefore, a good understanding of the underlying neurophysiological mechanism of tinnitus is crucial for early diagnosis and the development of disease-specific treatments against tinnitus.

A large body of neuroimaging studies has provided evidence that tinnitus is associated with functional and anatomical changes in several parts of the brain, including the auditory cortex, basal ganglia, prefrontal cortex, parahippocampal regions, and insula (Burton et al., 2012; Maudoux et al., 2012a,b; Chen et al., 2017; Hullfish et al., 2019; Berlot et al., 2020). However, it has been proposed that the unified percept of tinnitus can be considered an emergent property of dynamically changing networks (De Ridder et al., 2014). Furthermore, the cerebral cortex is organized into segregated complex networks that are specialized for processing and exchanging distinct forms of information (Buckner et al., 2009; Xiong et al., 2020). It is suggested that tinnitus is a complicated hearing impairment that is not only involved in the damage of isolated regions but also related to the brain network-level alternations. Therefore, it is imperative to explore brain function at the network level in tinnitus.

However, to date, only a few previous studies have examined tinnitus from the perspective of the brain network level. According to a study conducted by Davies et al. (2014), it was revealed that auditory network connectivity is not modified by the experience of tinnitus. The study also found altered functional connectivity (FC) in brain regions related to attention and emotional processing only in bothersome tinnitus. However, another previous study suggested that the tinnitus percept is not only linked to the activity in sensory auditory areas but is also associated with connectivity changes in non-auditory regions. This shows that there is a modification of cortical and subcortical FC in tinnitus encompassing attentional, mnemonic, and emotional networks (Maudoux et al., 2012b). Elsewhere, Schmidt et al. (2013) identified specific alterations in the connectivity of the default mode, dorsal attention, and auditory resting-state networks (RSN) due to tinnitus. This especially increased FC between limbic regions and auditory as well as dorsal attention RSNs in tinnitus participants (Schmidt et al., 2013). Furthermore, a study conducted by Leaver et al. (2016) presented a unique, atypical “tinnitus network” in patients with tinnitus and suggested that tinnitus pathophysiology involves crosstalk, and perhaps dysregulation, between frontostriatal and auditory–sensory regions. Recently, a resting-state functional magnetic resonance imaging (rs-fMRI) study that used a large sample size found that the connectivity patterns of the right executive control network, which is relevant for the perception of external stimuli, are mostly affected by the distress of patients with tinnitus (Kandeepan et al., 2019).

Although the previous studies provided valuable insights into the role of network interaction in the emergence of clinical tinnitus characteristics, they mostly focused on chronic tinnitus and did not analyze the interactions between each network. However, the patterns of brain networks in acute tinnitus still remain unclear. Tinnitus is usually associated with hearing loss. Approximately 75% of unilateral tinnitus and over 80% of bilateral tinnitus patients have a hearing loss in the standard audiogram detection with thresholds exceeding 20 dB (Wallhäusser-Franke et al., 2017). Therefore, studies on acute tinnitus patients with hearing loss (ATHL) may provide new insights into the investigation of the pathophysiological mechanism of acute tinnitus.

Independent component analysis (ICA), a data-driven method without prior experimental models or assumptions (McKeown et al., 1998), has been proven to be a helpful tool for the detection and isolation of various brain function networks (Davies et al., 2014; Leaver et al., 2016; Kandeepan et al., 2019; Li et al., 2020; Xing et al., 2020). Meanwhile, function network connectivity (FNC) is also a powerful way to assess interactions between RSNs that are based on the correlation between time courses of independent components (IC) (Wang et al., 2014; Qin et al., 2018). However, studies on inter-network connectivity changes in ATHL have not been reported. Therefore, investigations of the RSNs and FNC may offer more useful information to enhance the understanding of neural mechanisms underlying patients with acute tinnitus.

This study aimed to systematically investigate the intra- and inter-network connectivity alterations in ATHL. Interactions between brain networks were quantified using the temporal correlation of their spontaneous activity to estimate the group differences that could be associated with clinical characteristics. Two hypotheses were proposed in this study: first, abnormal FC within and between networks may exist in the ATHL group as compared with the HC group; second, these group differences would be associated with clinical characteristics.

## Materials and methods

### Subjects

A total of 40 patients were recruited from the Department of Otolaryngology, and a healthy group consisting of 40 participants was also recruited through online and print advertisements in the local community. The two groups were matched for age, gender, education, and handedness. All the patients had constant, unilateral tinnitus lasting less than 1 month with sensorineural hearing loss in the same ear, and they did not have tinnitus or hearing loss before. The hearing thresholds were assessed using pure-tone audiometry (PTA) at frequencies of 125, 250, 500, 1,000, 2,000, 4,000, and 8,000 Hz. It was evident that all the patients had hearing loss, defined as hearing thresholds of > 30 dB in one ear at frequencies from 0.125 to 8 kHz. Each participant in the HC group was confirmed to have a normal hearing level (hearing thresholds ≤ 20 dB at any tested frequency). In addition, exclusion criteria for this study, which were described in our previously published studies (Zhou et al., 2019, 2021), included the following: (a) ear diseases that impacted hearing condition (i.e., pulsatile tinnitus, hyperacusis, or Meniere’s disease); (b) a history of severe alcoholism, smoking, and head injury; (c) neurological or psychiatric illness such as stroke, Alzheimer’s disease, Parkinson’s disease, epilepsy, or major depression; (d) major medical illness such as cancer, anemia, or thyroid dysfunction; and (e) MRI contraindications. This study was approved by the Research Ethics Committee of Nanjing Medical University and written informed consent was obtained from all participants before the beginning of the study.

Moreover, the Tinnitus Handicap Inventory (THI), a self-reported tinnitus handicap questionnaire, provides assessments of tinnitus severity in all tinnitus patients with hearing. Before image scanning, all participants were asked to complete the Self-Rating Depression Scale (SDS) and Self-Rating Anxiety Scale (SAS) to evaluate their emotional states. Therefore, it was found that none of the participants had depression or anxiety, defined as overall scores < 50. The detailed clinical characteristics and demographics of all participants are listed in Table 1.

**TABLE 1:** Demographic and clinical characteristics of participants.

	Patients (*n* = 40)	Controls (*n* = 40)	*P*-value	ES
Age (years)	42.65 ± 11.92	46.08 ± 13.42	0.231	0.27
Gender (male/female)	18/22	16/24	0.653	–
Education (years)	12.18 ± 2.96	12.63 ± 3.27	0.521	0.14
Handedness (right/left)	40/0	40/0	1.000	–
Tinnitus laterality (right/left)	17/23	–	–	–
Duration (days)	7.43 ± 3.15	–	–	–
PTA of right ear (dB)	49.87 ± 15.95	17.68 ± 3.44	<0.001*	2.78
PTA of left ear (dB)	44.32 ± 13.85	18.14 ± 3.91	<0.001*	2.57
THI score	33.13 ± 8.96	–	–	–
SAS score	31.38 ± 5.12	25.48 ± 2.67	<0.001*	1.44
SDS score	30.35 ± 6.11	25.80 ± 2.03	<0.001*	0.99

Data are presented as mean ± SD. PTA, pure-tone audiometry; THI, Tinnitus Handicap Inventory; SAS, Self-Rating Anxiety Scale; SDS, Self-Rating Depression Scale; ES, effect size.

*P < 0.001 (independent-sample *t*-test, two-tailed) showed a statistical difference in hearing threshold between patients and HCs.

### MRI data acquisition

Imaging data were acquired using a 3.0 T MRI scanner (Ingenia, Philips Medical Systems, Netherlands) with an 8-channel receiver array head coil. Headphones and sponge pads were used to minimize scanner noise and head movement. During the scan, all the subjects were instructed to rest quietly with their eyes closed but to remain awake and avoid thinking about anything in particular. Structural images were acquired with a three-dimensional turbo fast echo T1WI sequence with high resolution as follows: repetition time (TR) = 8.1 ms; echo time (TE) = 3.7 ms; slices = 170; thickness = 1 mm; gap = 0 mm; flip angle (FA) = 8°; acquisition matrix = 256 × 256; and field of view (FOV) = 256 mm × 256 mm. The structural sequence was obtained in 5 min and 29 s. For rs-fMRI images, a gradient echo-planar imaging sequence was used with the following parameters: TR, 2,000 ms; TE, 30 ms; FA, 90°; the number of slices, 36; FOV, 220 × 220 mm 2; matrix size, 64 × 64; slice thickness, 4 mm; and total volume, 230; and this sequence required 8 min and 8 s. Finally, conventional MRI sequences, including axial T2WI and sagittal T2WI FLAIR sequences, were acquired to exclude intracranial organic lesions.

### MRI data preprocessing

Preprocessing of rs-fMRI data was performed using the toolbox of Data Processing and Analysis for Brain Imaging (DPABI V4.2^1^) (Yan et al., 2016), which is based on the Statistical Parametric Mapping software (Penny et al., 2007). For the data of each participant, the first 10 time points were discarded to ensure a steady state. Then, the remaining 220 images were slice-time corrected and realigned for head-motion correction. The participants who exhibited head motion > 2.0 mm translation or > 2.0° rotation were excluded from this study. The generated images were spatially normalized to the Montreal Neurological Institute (MNI) template with a resampling voxel size of 3 mm × 3 mm × 3 mm and then smoothed by convolution with a 6-mm full width at half maximum isotropic Gaussian kernel.

### Independent component analysis

#### Identification of resting-state networks

To obtain the different RSNs in this study, ICA analyses were performed using Group ICA of the fMRI toolbox (GIFT^2^) for all the participants. First, the estimated number of the ICs was determined using the minimum description length criteria, which was 29 for all the participants. Second, fMRI data were concatenated across all participants and then reduced to 29 components through principal component analysis, followed by IC estimation using the Infomax algorithm. This step was conducted using the ICASSO algorithm, which repeated the ICA analyses 100 times to ensure estimation stability. Finally, the group ICA (GICA) back-reconstruction method was used to generate subject-specific spatial maps and time courses, and hence the results were transformed into z-scores.

#### Intra-network functional connectivity analysis

Among the 29 components arising from ICA, 10 components (7 meaningful RSNs) were selected as the focus of subsequent analysis through visual inspection based on previous rs-fMRI studies (Bernas et al., 2018; Huang et al., 2020; Li et al., 2020; Xing et al., 2020). A one-sample *t*-test, which was corrected by a critical threshold with *p* < 0.01 (family-wise error correction, FWE), was performed on each RSN to determine the z-maps for each group. Then, two-sample *t*-tests were used to obtain the group differences of the z-maps of the RSNs. Group comparisons were restricted to the voxels within a union mask. The mask was generated by integrating regions of corresponding RSNs in both groups, which were obtained from one-sample *t*-test results. For group-level comparison, clusters passing a two-tailed Gaussian random field (GRF) correction with voxel-level *p* < 0.001 and cluster-level *p* < 0.005 were considered significant (age, gender, education, hearing level, SAS score, and SDS score were considered covariates).

#### Inter-network functional connectivity analysis

The FNC toolbox implanted in the GIFT software was employed to obtain temporal relationships between RSNs. Temporal band-pass filtering (0.00–0.1 Hz) of the imaging data was first carried out to reduce the influence of low-frequency drift and high-frequency physiological noise. The correlations between any two RSN time courses of each subject were then calculated. A 10 × 10 FNC matrix was later generated by calculating the Pearson correlation coefficient between the time courses of selected RSNs. Finally, a general linear model (GLM) was employed to analyze the group differences for each pair of RSNs between HC and ATHL (age, gender, education, hearing level, SAS score, and SDS score were considered covariates). The significance threshold was *p* < 0.001, uncorrected.

### Statistical analyses

Between-group differences in demographic variables were examined using independent two-sample *t*-tests for continuous variables and chi-square tests for categorical variables using the SPSS 22.0 software (IBM, Chicago, IL, United States), with a *P*-value of < 0.05 considered statistically significant. The tests of normality of the data distribution were determined using the Shapiro–Wilk tests, and a *P*-value of > 0.05 indicated that the experimental data were normally distributed. Cohen’s d was then used to describe the effect size (ES) of each clinical feature. Meanwhile, a two-sample *t*-test was conducted for RSNs analysis to obtain group differences, and the results were corrected for the GRF method (two-tailed, voxel-level *p* < 0.001, cluster-level *p* < 0.005).

Pearson correlation was used in this study to examine the relationship between FC in the RSNs/FNC and clinical features, including duration, THI, SDS, and SAS (statistical significance level *P* < 0.05, controlling for the effects of age, gender, education, and hearing level). During the current study, the voxel-level statistical analysis of RSNs was conducted using SPM12 (statistical parametric mapping) and the MATLAB function (MATLAB 2013a) was also used for FNC group comparison (*p* < 0.001, uncorrected). The Bonferroni correction for multiple comparisons was used for the correlation analysis in this study.

## Results

### Demographic and clinical information

Results of this study showed that there were no significant differences in the age, gender, or educational level (*P* > 0.05) of the participants in both patient and control groups (Table 1). During the auditory measurements, it was noted that all the patients with acute tinnitus exhibited unilateral hearing loss, whereas the participants in the HC group had a normal hearing level (*P* < 0.05). In addition, both SAS and SDS scores in the patient group were higher than those in the HC group (*P* < 0.05).

### Resting-state networks

**FIGURE 1:**
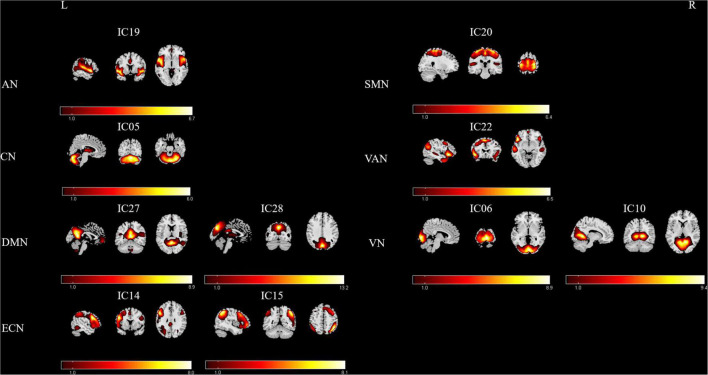
Relevant RSNs extracted from the group-level ICA. The spatial maps of 10 ICs were selected as the RSNs for further analysis. AN, auditory network; CN, cerebellum network; DMN, default mode network; ECN, executive control network; SMN, sensorimotor network; VAN, ventral attention network; VN, visual network. R, Right; L, Left.

After group ICA processing, 29 ICs were extracted from the fMRI data of all participants, and 10 components were selected as the RSNs. Subsequently, seven meaningful RSNs (Figure 1) were obtained, which was in accordance with previously reported research and included the following networks: The auditory network (AN; IC19) primarily encompassed the bilateral middle temporal gyrus, superior temporal gyrus, temporal pole, and insular. The sensorimotor network (SMN; IC20) was focused on the bilateral precentral and postcentral gyrus and the supplementary motor area. The cerebellum network (CN; IC5) included bilateral cerebellum hemispheres. The default-mode network (DMN; IC27+28) mainly included the precuneus/posterior cingulate cortex, inferior lateral parietal lobule, medial prefrontal cortex, superior frontal gyrus, and angular gyrus. The visual network (VN; IC6+10) was located in the middle occipital gyrus, superior occipital gyrus, temporal-occipital regions, and fusiform gyrus. The executive control network (ECN; IC14+15) also included the left lateral frontoparietal network (LFPN) and the right lateral frontoparietal network (RFPN). The LFPN along with RFPN showed similar spatial patterns, which were mainly focused on the middle frontal gyrus (MFG), inferior parietal lobule, superior parietal lobule, and angular gyrus. Furthermore, the ventral attention network (VAN; IC22) primarily involved the left and right superior temporal sulci, temporal poles, insula, middle frontal gyrus, and supplementary motor area.

### Intra-network connectivity differences

**FIGURE 2:**
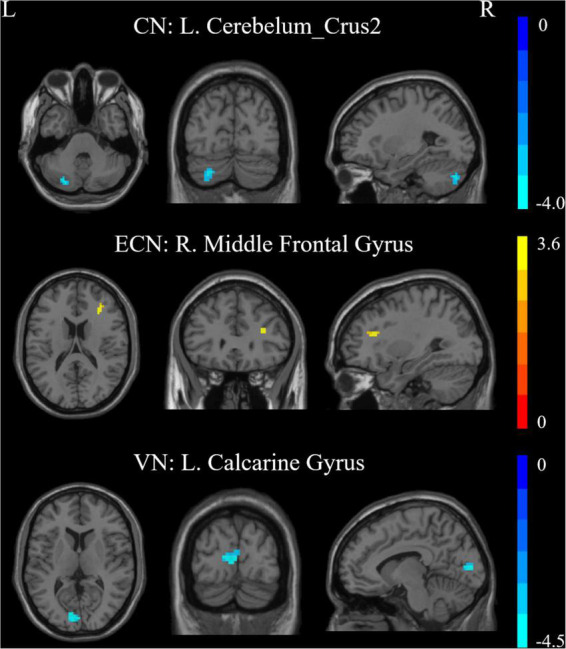
Intra-network connectivity differences within RSNs in the patients vs. controls. CN, cerebellum network; ECN, executive control network; VN, visual network; R, Right; L, Left.

Results of this study observed significant alterations in FC within 3 RSNs and between the patient and HC groups, including the CN, ECN, and VN (Figure 2 and Table 2). Furthermore, the ATHL group exhibited decreased FC within the CN (left cerebellum_crus2) and VN (left calcarine gyrus) as compared with the HC group. In addition, there was increased FC within the ECN (right MFG) in the patients as compared with the HCs. However, no significant differences were observed in FC within the DMN, SMN, DAN, and AN groups.

**TABLE 2:** Brain regions with significant difference connectivity within RSNs between patients and healthy controls.

	Brain region	Peak MNI coordinates	T score	Cluster size (voxels)
		(x, y, z)		
CN	L. Cerebelum_Crus2	−27, −78, −36	−3.9593	55
ECN	R. Middle Frontal Gyrus	30, 30, 18	3.5888	22
VN	L. Calcarine Gyrus	−9, −87, 9	−4.4746	46

The two-tailed GRF method was employed for multiple comparisons (voxel-level *p* < 0.001, cluster-level *p* < 0.005). L, Left; R, Right; MNI, Montreal Neurological Institute; CN, cerebellum network; ECN, executive control network; VN, visual network.

### Inter-network connectivity differences

**FIGURE 3:**
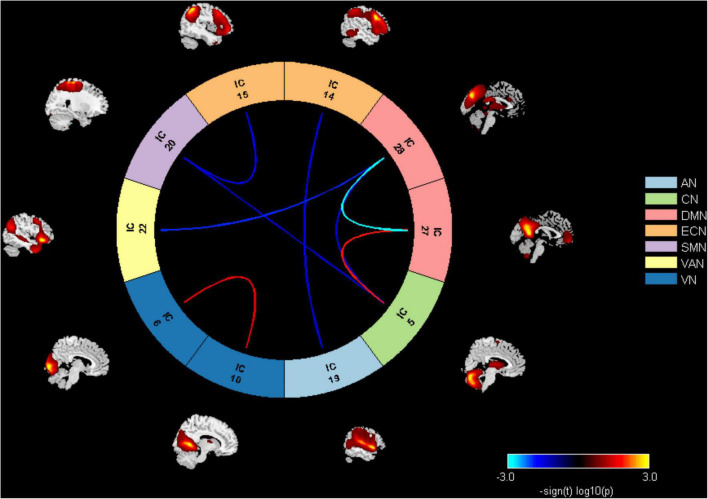
Inter-network connectivity differences between groups. AN, auditory network; CN, cerebellum network; DMN, default mode network; ECN, executive control network; SMN, sensorimotor network; VAN, ventral attention network; VN, visual network.

Results of the FNC analysis in this study showed that the patients with displayed aberrant network connectivity in AN, CN, ECN, SMN, VAN, and DMN as compared with the control groups (Figure 3). Specifically, the patient group showed decreased inter-network connectivity in the SMN (IC20)-CN (IC5), SMN (IC20)-ECN (IC15), DMN (IC28)-VAN (IC22), DMN (IC28)-CN (IC5), and ECN (IC14)-AN (IC19) connections. Meanwhile, significantly increased inter-network connectivity in the DMN (IC27)-CN (IC5) was also found in patients. Moreover, it was observed that there was a significantly decreased inter-network connection in the DMN (IC27)-DMN (IC28) in the ATHL group and also a significantly increased connection in the VN (IC6)-VN (IC10).

### Correlation analysis

**FIGURE 4:**
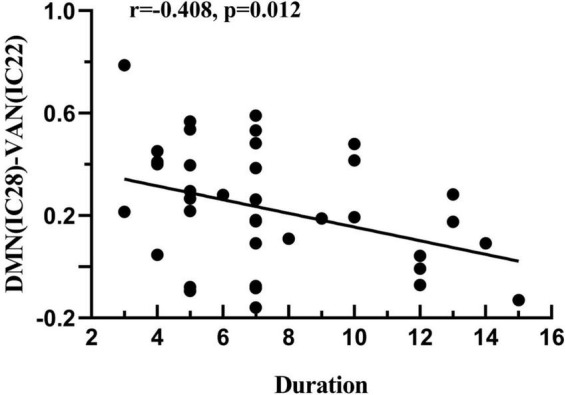
Correlation between the FNC coefficient and the clinical features in acute patients with hearing loss. DMN-VAN connection was found to be negatively correlated with duration (*r* = –0.408, *p* = 0.012).

Correlations were analyzed between the altered FC in the four RSNs and clinical data. However, it was found that there were no significant correlations in this correlation analysis. In addition, after computing the relationships between the FNC coefficients and clinical features in the ATHL group, it was found that the negative correlation with tinnitus duration was only demonstrated by the DMN-VAN connection, and this correlation survived after Bonferroni correction (*r* = −0.408, *P* = 0.012 < 0.05/4) (Figure 4).

## Discussion

To the best of our knowledge, this study is the first rs-fMRI study based on the ICA method to explore the intra- and inter-network FC as well as their relationship in tinnitus at an early stage. Furthermore, this study indicated abnormalities in several brain networks in the ATHL group as compared with the controls, including ECN, CN, and VN. Meanwhile, aberrant inter-network connectivity was observed in patients through FNC analysis.

The tinnitus participants in this study showed relatively low scores in the THI, SDS, and SAS tests. Even though the patients with ATHL showed higher scores in SDS and SAS than the healthy participants, the overall scores were still less than 50, which means that the ATHL group did not have a depression or anxiety state according to Zung’s research (Zung, 1971, 1986). Therefore, it is believed that these tinnitus-related features have less effect on patients with AT. Furthermore, the correlation analysis conducted in this study found no relationships between changes in intra- and inter-network FC and THI, SDS, and SAS scores, which is support the viewpoint given in this study. In contrast, tinnitus has long been associated with hearing impairments, and ruling out hearing loss as an alternative explanation for any observed effects is always an important methodological consideration in tinnitus research. Although we add hearing loss as a covariate during analysis, the confounding effect of hearing loss has not been satisfactorily addressed. More work is needed in this area such as studying a group with acute tinnitus without hearing loss would be incredibly useful.

The analysis of brain FC alteration within RSNs may elucidate the abnormal intrinsic interaction in a certain spatial pattern (Beckmann et al., 2005; De Luca et al., 2006). In this study, the ATHL group presented an increased FC in the right MFG for ECN. Furthermore, the ECN participates in many advanced cognitive tasks and plays an important role in adaptive cognitive control (McHugh et al., 2017). According to a study conducted by Rosemann and Thiel (2019) using fMRI, it was found that increased frontal activation was noted in patients with hearing loss, which possibly reflects an increased effort in executive function. Elsewhere, another study demonstrated that the activity of the central auditory pathway decreased as a result of hearing loss, resulting in compensatory increased activation in the ECN (Rutherford et al., 2018). Furthermore, it is believed that the ECN is involved in the allocation of top-down attentional resources (Fassbender et al., 2006). Functionally, the ECN is considered a “higher-order” network, as opposed to AN or SMN, which are considered “lower-order” networks (Power et al., 2011; Guldenmund et al., 2016). Modification of the functional coupling of the “higher-order” network with the “lower-order” network influences how the information is processed and whether the information is consciously perceived (Sadaghiani et al., 2009). The analysis of brain FC of human fMRI data revealed that sensory regions selectively process relevant information and are functionally connected with the ECN (Chadick and Gazzaley, 2011). Therefore, the processing of sensory cortical activity was greatly influenced by the top-down modulations from ECN. Results of disrupted inter-network for ECN-AN and ECN-SMN in this study showed that ATHL is associated with a modification of FC not only within the regions of ECN but also between regions belonging to different networks.

The DMN is activated at rest and hence shows reduced activity during task-related activities or when an executive function is required (Raichle et al., 2001). It is functionally involved in working memory and the interruption of the attention network also causes memory impairment (Veldsman et al., 2019; Xing et al., 2020). Therefore, the results of the hypo-connection for DMN-VAN in this study may indicate the impairment of memory and attention in the patient group. However, the findings still require more specific neuropsychological scales for verification. In addition, tinnitus duration in this study was negatively correlated with the DMN-VAN connection, indicating that the disrupted interaction between DMN and VAN may be related to the neuropathological changes in ATHL. In contrast, it has been shown that the attention network is responsible for top-down attention orientation and participates in exogenous attention orientation (Tripathy et al., 2017; Suo et al., 2021). The decreased DMN-VAN connectivity would mean that patients with tinnitus would probably already start to draw their attention inward toward their perception, and this change is correlated with tinnitus duration.

The cerebellum is mainly thought to be restricted to motor control and coordination, but growing evidence has suggested that the cerebellum may also have a vital role in sensory-perceptual processing (Konoike et al., 2012; Stoodley et al., 2012; Baumann et al., 2015; Xu et al., 2019). According to the results of previous studies, it has been reported that not only the temporal auditory areas of the cerebral cortex displayed activation during auditory stimulus but also specific areas in the cerebellum (Petacchi et al., 2005). Human and animal studies have demonstrated that various regions in the cerebellum such as tinnitus, hyperacusis, and hearing loss are activated in its contribution to hearing impairments (Stoodley and Schmahmann, 2009; Chen et al., 2015a). The findings of this study showed a decreased connectivity within the CN in the patient group. The results were consistent with the results of our previous work and a recent study focused on acute tinnitus, which showed reduced activity in the cerebellum (Zhou et al., 2019; Cai et al., 2020). In addition, a separate study conducted by Zhang et al. (2018) found enhanced and decreased connectivity between CN and other networks in unilateral hearing loss. This study also found disrupted connectivity in CN-SMN and CN-DMN, and these findings provide support for the cerebellum as a crucial node in patients with ATHL.

The calcarine cortex, which plays a significant role in the primary visual cortex, showed decreased FC in the ATHL group in this study, which is consistent with our previous study (Zhou et al., 2019). Some other neuroimaging studies have also found that patients with tinnitus exhibit reduced neural activity in the visual cortex (Burton et al., 2012; Chen et al., 2014, 2015b). Compensatory mechanisms in visual regions may be associated with phantom sound perception. In other words, sensory deprivation in the auditory modality results in the recruitment of the deprived modality by the visual modality (Bavelier et al., 2006; Dieterich et al., 2007). Furthermore, another possibility is that the visual system is “irrelevant” to processing the apparition of sounds in tinnitus.

The auditory network (AN) is likely to play a key role in the occurrence of the phantom sound of tinnitus. Structural and functional anomalies of the primary auditory cortex and secondary auditory regions have been found in chronic tinnitus (Cai et al., 2020). On the contrary, a study conducted by Davies et al. (2014) has demonstrated that there are no significant differences in the auditory cortical FC between patients with chronic tinnitus and healthy people. Results of this study also found no significant FC changes in auditory regions, which is consistent with our previous reports (Zhou et al., 2019, 2021). It was speculated that the inconsistent results obtained may be caused by several reasons: (1) All patients with tinnitus in this study are in the acute stage, so it may be a short time that there are no neuroplastic changes occurred in auditory regions; (2) tinnitus heterogeneity, such as the laterality, hearing level, and severity of tinnitus; and (3) different neuroimaging methods employed. Therefore, there is a need for more studies with more subgroups and different neuroimaging approaches to confirm the mechanism of AN in patients with acute tinnitus.

The current study had some limitations. Due to the relatively small sample size and a cross-sectional study design, the results have to be viewed with caution. Although this study was performed using strict inclusion and exclusion criteria, the influence of heterogeneity still exists (tinnitus laterality, degree of hearing loss, and depression or anxiety state). Therefore, there is a need for future studies with larger sample sizes and more subgroups, as well as using a longitudinal study design will be appropriate. In addition, this study performed limited brain networks. Furthermore, other networks may play an important role in the pathophysiology of acute tinnitus, such as the salience network and basal ganglia network. Exploring the dysfunction of the brain network level will also provide meaningful evidence for understanding the neural mechanism of acute tinnitus. Moreover, no meaningful attempt is made to either ensure that subjects in the two groups directed their attention similarly in the scanner or to assess afterward how they directed their attention. It may have some effects on attention or rest-related networks. Finally, although earphones were used to reduce the MR scanner noise in this study, the neural activity of the auditory pathway is likely to be influenced by scanner noise to a certain degree.

## Conclusion

In conclusion, this study indicated widespread alterations in intra- and inter-network connectivity in ATHL, suggesting that multiple large-scale network dysfunctions and interactions are involved in the early stage. Furthermore, our findings may provide new perspectives to understand the neuropathophysiological mechanism of acute tinnitus.

## Data availability statement

The original contributions presented in the study are included in the article/supplementary material, further inquiries can be directed to the corresponding author/s.

## Ethics statement

The studies involving human participants were reviewed and approved by the Research Ethics Committee of Nanjing Medical University. The patients/participants provided their written informed consent to participate in this study.

## Footnotes

1
http://rfmri.org/dpabi

2
http://icatb.sourceforge.net/
